# Association Between Left Ventricular Diastolic Dysfunction, Systemic Inflammation, and Gastrointestinal Symptoms in HIV-Positive Patients on Antiretroviral Therapy

**DOI:** 10.3390/diseases12120313

**Published:** 2024-12-03

**Authors:** Madalina-Ianca Suba, Bogdan Hogea, Ahmed Abu-Awwad, Voichita Elena Lazureanu, Ovidiu Rosca, Daniela Gurgus, Sorina Maria Denisa Laitin, Alina Abu-Awwad

**Affiliations:** 1 Doctoral School, “Victor Babes” University of Medicine and Pharmacy, Eftimie Murgu Square, No. 2, 300041 Timisoara, Romania; madalina.suba@umft.ro; 2 Clinical Hospital for Infectious Diseases and Pneumophthisiology “Doctor Victor Babes”, 300310 Timisoara, Romania; lazureanu.voichita@umft.ro (V.E.L.); laitin.sorina@umft.ro (S.M.D.L.); 3 Department XV—Discipline of Orthopedics-Traumatology, “Victor Babes” University of Medicine and Pharmacy, 300041 Timisoara, Romania; hogea.bogdan@umft.ro (B.H.); ahm.abuawwad@umft.ro (A.A.-A.); 4 Research Center Professor Doctor Teodor Sora, “Victor Babes” University of Medicine and Pharmacy, 300041 Timișoara, Romania; 5 Department XIII, Discipline of Infectious Diseases, “Victor Babes” University of Medicine and Pharmacy, Eftimie Murgu Square 2, 300041 Timisoara, Romania; 6 Department of Balneology, Medical Recovery and Rheumatology, Family Discipline, Center for Preventive Medicine, “Victor Babes University of Medicine and Pharmacy, 300041 Timisoara, Romania; gurgus.daniela@umft.ro; 7 Discipline of Epidemiology, “Victor Babes” University of Medicine and Pharmacy Timisoara, 300041 Timisoara, Romania; 8 Ist Clinic of Obstetrics and Gynecology, “Pius Brinzeu” County Clinical Emergency Hospital, 300723 Timisoara, Romania; alina.abuawwad@umft.ro; 9 Department of Obstetrics and Gynecology, Faculty of Medicine, “Victor Babes” University of Medicine and Pharmacy, 300041 Timisoara, Romania

**Keywords:** HIV, ART, LVDD, systemic inflammation, gastrointestinal complications, inflammatory biomarkers, TNF-alpha, cardiovascular comorbidities

## Abstract

Background/Objectives: Despite advancements in antiretroviral therapy (ART), HIV-positive individuals face heightened risks of cardiovascular and gastrointestinal (GI) complications, often linked to persistent systemic inflammation. Left ventricular diastolic dysfunction (LVDD), prevalent in HIV patients, exacerbates this inflammatory state and may contribute to worsened GI symptoms. This study aims to explore the association between LVDD, systemic inflammation, and gastrointestinal symptoms in HIV-positive patients undergoing ART. The primary objective is to analyze how LVDD contributes to the inflammatory burden and its impact on gastrointestinal health in this population. Methods: This cross-sectional study included 320 participants divided into three groups: HIV-positive with LVDD (n = 80), HIV-positive without LVDD (n = 120), and HIV-negative controls (n = 120). Levels of inflammatory biomarkers—CRP, IL-6, TNF-α, fibrinogen, IL-1β, IFN-γ, and D-dimer—were measured, and GI symptoms were assessed. Echocardiographic evaluations were performed to determine LVDD presence and severity, while multivariate logistic regression identified predictors of GI complications. Results: Patients in the HIV + LVDD group exhibited significantly elevated levels of TNF-α, CRP, and D-dimer compared to other groups, correlating with higher incidences of nausea, diarrhea, and abdominal pain. TNF-α emerged as the strongest predictor of GI symptoms, underscoring its role in the pathophysiology linking cardiovascular and GI distress in this population. Persistent inflammation and coagulation abnormalities in the ART + LVDD group suggest that ART alone may not fully mitigate these complications. Conclusions: Our findings emphasize the compounded inflammatory burden in HIV patients with LVDD, highlighting the need for integrated approaches that address both cardiovascular and GI symptoms. Anti-inflammatory therapies targeting specific biomarkers like TNF-α could improve clinical outcomes, supporting a more comprehensive strategy to managing HIV-related comorbidities beyond viral suppression.

## 1. Introduction

Human immunodeficiency virus (HIV) infection remains a primary global health concern, affecting millions of individuals worldwide [1]. Despite the significant advances in antiretroviral therapy (ART), which have transformed HIV from a fatal disease to a manageable chronic condition, patients with HIV continue to experience a wide range of complications due to persistent immune activation and systemic inflammation [2]. Even under practical ART, this chronic inflammatory state is associated with various comorbidities, particularly affecting cardiovascular [3] and gastrointestinal (GI) [4] systems. Such complications not only contribute to morbidity and mortality but also significantly impair the quality of life in individuals living with HIV.

Persistent inflammation in HIV-infected patients can accelerate the progression of cardiovascular diseases (CVDs) and is particularly implicated in the development of left ventricular diastolic dysfunction (LVDD) [5]. LVDD occurs when the heart’s left ventricle has difficulty relaxing and filling correctly, leading to increased pressure [6]. This dysfunction often progresses silently but is closely related to the risk of developing heart failure with preserved ejection fraction (HFpEF), a type of heart failure that is challenging to manage and is associated with significant morbidity [7]. Studies have shown that HIV-positive individuals exhibit higher rates of LVDD compared to the general population, likely due to a combination of immune activation, systemic inflammation, and metabolic alterations induced by both the infection and long-term antiretroviral therapy (ART) [5,8]. Notably, research indicates that while HIV patients have impaired diastolic function, this impairment is not exacerbated by ART.

In addition to cardiovascular issues, GI complications are frequently observed in HIV-infected patients. These can range from malabsorption and nutrient deficiencies to more severe conditions such as enteropathy and inflammatory bowel disease-like symptoms. GI complications disrupt the digestive process and further aggravate systemic inflammation, creating a feedback loop that impacts other organ systems, including the cardiovascular system [9]. Although the link between HIV, systemic inflammation, and each of these individual complications (LVDD and GI issues) has been studied, there is limited research exploring how LVDD and GI complications may interact within the inflammatory context of HIV.

This study aims to explore the association between left ventricular diastolic dysfunction (LVDD), systemic inflammation, and gastrointestinal symptoms in HIV-positive patients undergoing antiretroviral therapy (ART). The primary objective is to analyze how LVDD contributes to the inflammatory burden and its impact on gastrointestinal health in this population.

Understanding this complex interaction between cardiac dysfunction and GI health in the context of HIV is critical for advancing clinical care. If a significant association is identified, these findings could inform a more integrated approach to managing HIV-related comorbidities. Such an approach would go beyond viral suppression and consider the systemic and interconnected nature of inflammation-driven complications in HIV, potentially leading to new treatment targets, improved risk stratification, and more personalized, holistic patient care.

## 2. Materials and Methods

### 2.1. Study Design and Population

This study was designed as a cross-sectional, observational analysis to investigate the association between left ventricular diastolic dysfunction (LVDD), systemic inflammation, and gastrointestinal (GI) complications in HIV-positive individuals undergoing antiretroviral therapy (ART). The study population was divided into three distinct groups to enable comparative analysis across varying levels of HIV and cardiovascular status.

The study population was divided into three groups for comparative analysis: the control group (Group 1), which included 120 HIV-negative individuals without cardiovascular disease, served as a baseline for inflammatory biomarkers and GI health, providing reference data to assess normal levels of these parameters in individuals without HIV or cardiac issues. The HIV-positive group (Group 2) consisted of 120 HIV-positive patients undergoing ART who did not exhibit left ventricular diastolic dysfunction (LVDD), allowing for focused analysis of ART-related inflammatory responses and GI side effects without significant cardiovascular impairment. Finally, the HIV + LVDD group (Group 3) comprised 80 HIV-positive patients on ART who also had LVDD, enabling an evaluation of how LVDD might influence both systemic inflammation and GI complications in HIV-positive individuals relative to the other two groups, given LVDD’s increasing prevalence among this population and its potential role in exacerbating multi-organ inflammatory processes.

All participants in the HIV-positive cohorts (HIV + LVDD and HIV+ non-LVDD) were on next-generation ART regimens, including integrase strand transfer inhibitors (Dolutegravir, 50 mg once daily; Bictegravir, 50 mg once daily as part of the fixed-dose combination Biktarvy) and nucleoside reverse transcriptase inhibitors (Tenofovir alafenamide, 25 mg once daily; Emtricitabine, 200 mg once daily), either as single agents or in fixed-dose combinations (e.g., Biktarvy: Bictegravir 50 mg, Emtricitabine 200 mg, Tenofovir alafenamide 25 mg). These regimens, widely adopted in modern HIV care for their safety and association with reduced systemic inflammation, were selected to minimize variability linked to older ART protocols and to focus on the specific contributions of LVDD to inflammatory and gastrointestinal outcomes.

Demographic and clinical variables such as age, gender, body mass index (BMI), and existing comorbidities were recorded across all groups to ensure robust and accurate comparisons. This structured division enabled targeted evaluation of the combined impact of HIV, ART, and LVDD on inflammatory and gastrointestinal outcomes.

Participants were selected from a large, urban health center specializing in infectious disease and cardiovascular care, specifically the “Dr. Victor Babeș Clinical Hospital for Infectious Diseases and Pneumophthisiology” in Timișoara, Romania. A total of 320 individuals were enrolled in this study, distributed as follows: 80 HIV-positive patients with LVDD (HIV + LVDD group), 120 HIV-positive patients without LVDD (HIV group), and 120 controls (control group). Biological and clinical data were collected at enrollment and six months later, covering 10 years from January 2013 to January 2023.

### 2.2. Inclusion and Exclusion Criteria

Inclusion Criteria

- Adults with a confirmed diagnosis of HIV who demonstrated consistent ART adherence over the past year, as verified by treatment records.
- HIV-positive status confirmed through both serological and PCR testing, with documentation in the patient’s medical file.
- Stable antiretroviral therapy (ART) regimen for at least 6 months before enrollment, without recent treatment modifications.
- Comprehensive medical records, including relevant laboratory, imaging, and clinical data necessary for cardiovascular and gastrointestinal evaluation, are available.
- Recent laboratory stability, with CD4 count, viral load, and other relevant immune markers maintained within stable ranges for at least the past 3 months.
- Clear understanding of the study protocol and the ability to provide informed consent.
- Absence of any known chronic gastrointestinal conditions or disorders unrelated to HIV infection or its treatment.
- No significant cardiovascular events, such as heart attack or stroke, were documented in the last 12 months.
- No clinical history of left ventricular diastolic dysfunction (LVDD) or other significant cardiac dysfunctions predating the HIV diagnosis.
- Body mass index (BMI) within a moderate range (18.5–30 kg/m^2^), excluding extremes that could introduce additional risk factors.

Exclusion Criteria

- Patients outside the age range of 21 to 65 years were excluded to reduce variability related to extremes of age in the study cohort.
- Recent initiation of ART (within the past 6 months) or records indicating non-adherence or inconsistent ART use within the past year.
- Active or chronic liver disease, including conditions like hepatitis B or C coinfections, cirrhosis, or significant hepatic impairment.
- Chronic renal impairment, particularly with an estimated glomerular filtration rate (eGFR) below 45 mL/min, to avoid confounding effects from renal-related inflammation.
- Pregnant or breastfeeding participants due to potential physiological changes that may affect inflammatory and cardiovascular markers.
- Recent major surgery (within the last 3 months) that could influence inflammatory or immune responses, thus confounding this study’s focus.
- The presence of autoimmune or other chronic inflammatory diseases (e.g., lupus, rheumatoid arthritis) that require immunosuppressive treatment.
- Use of corticosteroids, NSAIDs, or other immunosuppressive drugs within 3 months of enrollment, which may interfere with baseline inflammation markers.
- Cognitive impairment, severe psychiatric conditions, or other barriers that would hinder informed consent or adherence to study requirements.

### 2.3. Clinical and Laboratory Assessments

All participants underwent comprehensive echocardiography following a standardized protocol to evaluate cardiac function. The presence and severity of left ventricular diastolic dysfunction (LVDD) were assessed according to the American Society of Echocardiography (ASE) guidelines, using key indicators such as the E/A ratio, E/e’ ratio, and left atrial volume index [10]. Based on filling pressures and diastolic function parameters, only LVDD of Grade I and II severity was included in this study.

Grade I LVDD, also known as mild dysfunction, is characterized by impaired left ventricle relaxation but generally preserved filling pressures, often asymptomatic. Grade II LVDD, or moderate dysfunction, involves pseudonormal filling patterns with moderately elevated filling pressures and is more likely to present with clinical symptoms [11]. This study focused on Grades I and II because these stages represent early to moderate diastolic dysfunction, where systemic inflammation and GI symptoms can still be examined without the confounding effects of severe cardiac compromise. Certified cardiologists performed all echocardiographic evaluations, remaining blinded to each patient’s GI and inflammatory status.

Gastrointestinal complications were assessed based on patient history, clinical symptoms, and relevant laboratory tests, including markers of inflammation and malabsorption. Symptoms include diarrhea (three or more loose or watery stools per day for at least two consecutive days). Diarrhea was assessed using the patient’s medical history and symptoms reported during clinical visits, without classifying episodes by severity or type; abdominal pain, nausea, and weight loss were recorded, with additional assessments for nutrient malabsorption and intestinal permeability where necessary. Upper GI endoscopy or colonoscopy was performed when clinically indicated to confirm specific GI abnormalities.

### 2.4. Statistical Analysis

The statistical analysis for this study was performed using GraphPad Prism 6 software. For continuous variables, including age, BMI, ART duration, CD4 cell count, and inflammatory biomarker levels (CRP, IL-6, TNF-α), one-way ANOVA (most of the continuous variables were parametric) was used to compare the three groups (HIV-negative controls, HIV-positive without LVDD, and HIV-positive with LVDD). Post hoc Bonferroni tests were conducted to identify pairwise differences between groups. For categorical variables, such as gender, the presence of LVDD, and the incidence of gastrointestinal (GI) symptoms (e.g., nausea, diarrhea, abdominal pain, bloating), Chi-square tests were used to evaluate differences in proportions among the groups.

The *p*-values presented in the tables reflect comparisons across all three groups. Significant findings were further explored to determine whether differences existed specifically between the two HIV-positive groups. Additionally, Pearson correlation coefficients were calculated to assess the relationships between inflammatory biomarkers and the severity of GI symptoms. Lastly, multivariate logistic regression analysis was applied to identify independent predictors of GI complications, with odds ratios (ORs), 95% confidence intervals (CIs), and *p*-values reported for each predictor. All analyses were considered statistically significant at *p* < 0.05.

### 2.5. Ethical Consideration

Patient medical records were securely stored in a database compliant with data protection laws to ensure confidentiality and protect personal information. Access to these records was permitted only after obtaining informed consent from each participant, thus respecting patient confidentiality and upholding their rights throughout this study.

All procedures in this research adhered strictly to the ethical standards set by the relevant institutional and national human experimentation committees. They were also consistent with the principles of the Declaration of Helsinki (1975), including its most recent update in 2013. Informed consent was obtained from each participant before inclusion in this study, ensuring they were fully informed about this study’s nature, purpose, and scope.

The research protocol received formal approval from the hospital’s Ethics Committee, with approval number 8947/28 September 2018. This approval verified compliance with all applicable ethical guidelines and safeguarded the protection and respect of all participants involved in this study.

## 3. Results

Table 1 summarizes the study groups’ demographic and clinical characteristics. No significant differences exist in age, gender distribution, or (body mass index) BMI between the control, (antiretroviral therapy) ART, and ART + LVDD (left ventricular diastolic dysfunction) groups (*p* > 0.05). However, CD4 counts were significantly higher in the control group compared to the ART and ART + LVDD groups (*p* < 0.001), with the ART + LVDD group showing the lowest levels. Similarly, viral load was undetectable in the control group but significantly elevated in the ART and ART + LVDD groups, with pairwise comparisons showing statistical significance (*p* < 0.05). In addition to the demographic and clinical characteristics, Table 1 also includes a section detailing the antiretroviral therapy (ART) regimens administered to the ART and ART + LVDD groups. This information was included to provide transparency regarding the standardized treatment protocols used across these groups. The control group did not receive ART, as these participants were HIV-negative. By including these details, we aim to ensure that any potential variability due to ART regimens is accounted for in the study design. While ART regimens effectively reduce viral replication, their role in persistent inflammation and adverse gastrointestinal effects remains under investigation.

The biomarker levels before and six months after ART initiation showed significant global differences among the three groups (control, ART, and ART + LVDD) for all measured parameters (*p* < 0.001, ANOVA). Post hoc Bonferroni tests revealed that CRP, IL-6, TNF-α, fibrinogen, IL-1β, IFN-γ, and D-dimer levels were significantly higher in the ART and ART + LVDD groups compared to the control group (*p* < 0.05 for all pairwise comparisons), with the ART + LVDD group consistently exhibiting the highest values. Significant differences were also observed between the ART and ART + LVDD groups for most biomarkers, particularly TNF-α and D-dimer, underscoring the additional inflammatory burden associated with LVDD (Table 2).

After six months of antiretroviral therapy (ART), gastrointestinal symptoms such as nausea, diarrhea, abdominal pain, and bloating were significantly more frequent in the ART and ART + LVDD groups compared to the control group (Table 3). Nausea affected 41.66% of ART patients and 56.25% of those with LVDD, while diarrhea was reported in 37.5% and 55%, respectively, with both being significantly higher than in the control group (*p* < 0.001). Similarly, abdominal pain and bloating were more prevalent in the ART and ART + LVDD groups compared to controls, with all pairwise differences statistically significant. These findings highlight a clear link between ART, LVDD, and GI side effects, with the highest symptom burden in the ART + LVDD group.

Table 4 provides an overview of cardiac and inflammatory results for Group 3, comprising HIV-positive patients on ART with Grade 1 (mild) and Grade 2 (moderate) left ventricular diastolic dysfunction (LVDD). Key cardiac parameters, including the E/A ratio, E/e’ ratio, left atrial volume index, and ejection fraction, were assessed using ASE guidelines. Significant differences were observed between the two grades, with Grade 2 LVDD patients demonstrating higher E/e’ ratios and left atrial volume indices, reflecting increased ventricular filling pressures and more severe diastolic dysfunction. Moreover, a more significant proportion of Grade 2 patients exhibited clinical symptoms, with nausea (57.14%) and diarrhea (45.71%) being notably more prevalent compared to Grade 1 patients. Elevated inflammatory markers, such as TNF-α and IL-6, were also significantly associated with Grade 2 LVDD, further emphasizing the role of systemic inflammation in disease progression. These findings highlight the impact of LVDD severity on both cardiac function and systemic symptomatology within the HIV-positive cohort.

Table 5 presents Pearson correlation coefficients, illustrating significant associations between inflammatory biomarkers (CRP, IL-6, TNF-α, fibrinogen) and gastrointestinal symptoms (nausea, diarrhea, abdominal pain, bloating). The table includes data from all study participants, with the analyzed groups being the three previously mentioned: control, ART, and ART + LVDD (n = 320). These correlations highlight a strong link between elevated inflammatory markers and GI symptoms, particularly for TNF-α (r = 0.68 for nausea, r = 0.63 for diarrhea) and CRP (r = 0.65 for nausea, r = 0.60 for diarrhea), all with *p* < 0.01.

Table 6 presents the results of a multivariate logistic regression analysis aimed at identifying independent predictors of gastrointestinal (GI) complications in HIV-positive participants. The analysis includes both the ART and ART + LVDD groups (n = 320), focusing on factors associated with these complications within the HIV population. Variables analyzed include inflammatory biomarkers such as CRP, IL-6, TNF-α, fibrinogen, IL-1β, IFN-γ, and D-dimer, alongside age, BMI, and gender. Each parameter is reported with its odds ratio (OR), 95% confidence interval (CI), and *p*-value to evaluate its statistical significance as a predictor.

(*) statistically significant results.

The analysis highlights that elevated levels of CRP, IL-6, TNF-α, and fibrinogen are strong predictors for GI symptoms, with high OR values and narrow confidence intervals supporting their significance. For example, TNF-α had an OR of 1.82 (95% CI: 1.45–2.31, *p* < 0.0001), indicating a robust association with GI complications. CRP (OR 1.75) and IL-6 (OR 1.68) similarly showed strong predictive values for symptom occurrence.

Moreover, IL-1β, IFN-γ, and D-dimer were also identified as independent predictors of GI complications, emphasizing the role of systemic inflammation in the onset of these adverse effects. The findings underscore that heightened inflammatory markers, particularly in patients with HIV and LVDD, are closely linked to the development of GI symptoms.

Table 7 presents the results of logistic regression analysis, highlighting predictive factors for gastrointestinal (GI) symptoms among HIV+ and HIV + LVDD patients. Inflammatory markers such as TNF-α (OR = 1.82 for HIV+ and OR = 2.25 for HIV + LVDD) and CRP (OR = 1.75 for HIV+ and OR = 2.10 for HIV + LVDD) were the strongest predictors, indicating a significant association between systemic inflammation and GI symptoms, with an exacerbating effect of LVDD. Similarly, IL-6 and fibrinogen showed higher odds ratios in the HIV + LVDD group, emphasizing the additional impact of diastolic dysfunction on the severity of GI symptoms. Gender (female), age, and BMI were included in the analysis for both groups. It shows no statistically significant association with gastrointestinal symptoms in either the HIV+ group or the HIV + LVDD group. The significant *p*-values (<0.001 for most predictors) suggest a robust correlation between inflammatory markers and GI symptoms. The increased odds ratios in the HIV + LVDD group reflect a combined effect of inflammation and cardiac dysfunction on the gastrointestinal tract, underscoring the need for integrated approaches to manage these comorbidities.

## 4. Discussion

This study explores the intersection of systemic inflammation, cardiovascular dysfunction, and gastrointestinal (GI) outcomes in HIV-positive patients on antiretroviral therapy (ART), particularly those with left ventricular diastolic dysfunction (LVDD).

Our findings confirm that systemic inflammation persists in HIV-positive individuals on ART, particularly in those with LVDD, as evidenced by elevated CRP, IL-6, and TNF-α, consistent with prior research [12,13]. This inflammatory state contributes to cardiovascular and GI dysfunction [14].

Patients with LVDD exhibited the highest levels of TNF-α and CRP, aligning with prior findings that link systemic inflammation to myocardial fibrosis and diastolic dysfunction [15,16]. Elevated IL-6 and IL-1β levels further emphasize the role of ongoing cardiovascular stress in this population [17].

Markers of coagulation, such as D-dimer and fibrinogen, were significantly elevated in the ART + LVDD group, highlighting their contribution to cardiovascular and gastrointestinal complications [18,19,20,21].

Systemic inflammation, particularly in LVDD patients, may exacerbate gastrointestinal symptoms by impairing microcirculation and mucosal integrity, consistent with prior research [22,23,24,25,26]. TNF-α, in particular, may mediate GI symptoms due to its pro-inflammatory effects on GI mucosa [27], promoting both inflammation and permeability; this may explain the higher prevalence of GI symptoms in the ART + LVDD group.

Our analysis indicates that TNF-α correlates more strongly with GI symptoms than other inflammatory markers [28], underscoring TNF-α’s role in the pathophysiological link between cardiovascular dysfunction and GI distress in HIV patients. Prior research has shown that TNF-α enhances microbial translocation and perpetuates GI tract inflammation in chronic inflammatory conditions [29]. Mechanistically, TNF-α upregulates GI permeability and may stimulate enteric nerves, contributing to nausea and abdominal pain [30]. LVDD-induced congestion may further compromise gut perfusion, creating a hypoxic environment that worsens inflammation and GI discomfort [31].

Although ART effectively reduces systemic inflammation by suppressing viral replication, its impact on GI symptoms remains mixed [32]. While ART reduces HIV-related immune activation, specific regimens, particularly those containing protease inhibitors, are associated with gut microbiota dysbiosis and mucosal immune disruption, which can lead to chronic GI symptoms [33].

The inclusion of modern ART regimens, particularly Biktarvy and TAF/Emtricitabine/Dolutegravir, in this study reflects the current standard of care for HIV treatment. These regimens are known for their efficacy and relatively favorable side effect profiles, which minimized variability linked to older ART protocols. However, the potential influence of ART on gastrointestinal symptoms, such as those observed in our study, may be mediated through gut microbiota alterations or by enhancing pro-inflammatory states. This highlights the importance of selecting ART regimens carefully to balance efficacy with tolerability, especially in patients with comorbid conditions such as LVDD.

In our study, GI symptoms persisted for six months post-ART initiation, particularly among those with LVDD, suggesting that ART may inadvertently exacerbate GI distress in some patients by altering gut microbiota or enhancing pro-inflammatory states [34]. This ART-related pro-inflammatory environment could combine with LVDD-associated circulatory stress to worsen GI health through mechanisms affecting gut motility, absorption, and permeability [35].

The persistent elevation of CRP, IL-6, and fibrinogen in the ART + LVDD group underscores the compounded inflammatory burden in these patients [36]. Studies have shown that ART reduces viral load and immune activation but does not fully resolve the heightened inflammatory state associated with cardiovascular comorbidities [16]. Our results are consistent with studies, indicating that residual inflammation likely persists due to complex interactions between ART’s metabolic effects, ongoing immune activation, and cardiovascular dysfunction [3]. This compounded inflammatory response may promote adverse GI outcomes by impairing microvascular function, increasing oxidative stress, and driving endothelial dysfunction across organ systems [37].

Logistic regression identified TNF-α, CRP, IL-6, fibrinogen, and D-dimer as significant predictors of GI symptoms, highlighting the interplay between inflammation and coagulation in this context [38,39,40,41,42].

ORs for inflammatory biomarkers were consistently higher in the HIV + LVDD group, particularly for TNF-α and CRP, highlighting the exacerbating effect of LVDD on systemic inflammation and GI outcomes.

Additionally, other markers such as IL-6 and fibrinogen followed a similar pattern, with elevated ORs in the HIV + LVDD group compared to the HIV+ group, reflecting their role in both inflammation and coagulation pathways. Age and BMI also emerged as independent predictors, highlighting the contribution of patient-specific factors to the worsening of GI symptoms. These findings emphasize the compounded inflammatory and metabolic burden in individuals with both HIV and LVDD, which may exacerbate endothelial dysfunction and microvascular complications, particularly in the gastrointestinal tract.

In our multivariate logistic regression, gender, BMI, and age were included as covariates to ensure a comprehensive evaluation of factors potentially influencing gastrointestinal (GI) symptoms. Among these, BMI and age emerged as significant predictors, indicating their role in exacerbating GI outcomes in HIV+ patients, particularly those with LVDD. In contrast, gender did not show a significant association with GI symptoms in either the HIV+ group or the HIV + LVDD group. These results suggest that the observed GI outcomes are predominantly influenced by systemic inflammation, metabolic factors, and cardiovascular dysfunction, rather than gender differences. However, subtle sex-based biological variations, such as hormonal or immune responses, may warrant further investigation in larger cohorts.

This analysis reinforces the hypothesis that LVDD correlates with systemic inflammation and acts as a modifier, intensifying the association between elevated inflammatory markers and GI morbidity. Targeted interventions addressing systemic inflammation, particularly TNF-α and CRP, may be beneficial in mitigating GI symptoms in this high-risk subgroup.

Clinical implications and future directions

The implications of our findings highlight the need for an integrated approach to managing HIV-positive patients with cardiovascular complications. The persistent systemic inflammation observed in the ART + LVDD group suggests that standard ART may not be sufficient for patients with both HIV and cardiovascular dysfunction. Anti-inflammatory therapies, possibly targeting TNF-α specifically, could offer additional benefits for these patients by alleviating cardiovascular and GI symptoms.

Our findings also emphasize the importance of routine GI assessments for HIV patients with cardiovascular comorbidities. Regular monitoring of GI symptoms could facilitate early intervention, potentially improving patient adherence to ART and overall quality of life. Given the established link between systemic inflammation and GI health, incorporating anti-inflammatory or probiotic therapies to address gut dysbiosis may benefit HIV patients with concurrent cardiovascular issues.

Limitations and Strengths of this Study

One limitation of our study is its cross-sectional design, which limits the ability to draw causal inferences. Future longitudinal studies could better elucidate the cause-and-effect relationship between cardiovascular dysfunction, systemic inflammation, and GI symptoms in HIV patients. Additionally, this study’s reliance on specific inflammatory markers may not capture the entire inflammatory profile of each patient. Expanding biomarker panels in future research could provide a more comprehensive view of inflammation in these patients. Specifically, future studies could include additional biomarkers such as vascular endothelial growth factor (VEGF) for angiogenesis, monocyte chemoattractant protein-1 (MCP-1) for immune cell recruitment, and markers of oxidative stress like malondialdehyde (MDA) and superoxide dismutase (SOD). These markers could offer deeper insights into the inflammatory and metabolic pathways influencing both cardiovascular and gastrointestinal outcomes in HIV-positive individuals.

Despite these limitations, our study has several strengths, including a well-defined patient cohort and multivariate analysis to control for potential confounding variables. Our study’s focus on LVDD as a specific cardiovascular comorbidity also addresses a gap in the literature, as much of the existing research on cardiovascular health in HIV does not explore LVDD about GI symptoms.

## 5. Conclusions

This study provides important insights into the associations between systemic inflammation, cardiovascular dysfunction, specifically LVDD, and GI symptoms in HIV-positive individuals undergoing ART. Our findings highlight that patients with both HIV and LVDD exhibit a compounded inflammatory state, as evidenced by elevated levels of markers such as TNF-α, CRP, IL-6, and D-dimer. This sustained inflammatory response, which persists even with effective ART, likely contributes to the worsening of GI symptoms, including nausea, diarrhea, and abdominal pain, further impacting quality of life.

This study highlights TNF-α as a promising biomarker for GI symptoms in this population, emphasizing its potential role in the pathophysiological connection between cardiovascular dysfunction and GI distress in HIV patients. Elevated CRP and D-dimer levels were also significantly associated with GI symptoms, supporting the hypothesis that a coagulation–inflammation interplay may worsen microvascular function and contribute to mucosal disruption in the GI tract. Further studies are needed to validate these findings and explore the potential of these biomarkers as indicators.

Clinically, these findings suggest that standard ART may not fully address the inflammatory complications associated with cardiovascular dysfunction in HIV-positive individuals. Patients with concurrent LVDD may benefit from additional anti-inflammatory interventions, which could target specific markers such as TNF-α to reduce both cardiovascular and GI morbidity. The data also suggest that routine GI symptom monitoring is essential in managing HIV patients with cardiovascular comorbidities, as early intervention may improve both treatment adherence and overall quality of life.

In conclusion, our study emphasizes the importance of an integrated, multidisciplinary approach in managing HIV-related comorbidities. By addressing the systemic inflammation that links cardiovascular and GI complications, healthcare providers could optimize treatment outcomes in HIV patients with complex inflammatory profiles, paving the way for more personalized, holistic care strategies.

## Figures and Tables

**Table 1:** Clinic and demographic characteristics of the three groups.

Characteristic	Control Group (n = 120)	ART Group (n = 120)	ART + LVDD Group (n = 80)	Global *p*-Value (ANOVA)	Control vs. ART (*p*)	Control vs. ART + LVDD (*p*)	ART vs. ART + LVDD (*p*)
Age (years)	35 ± 8	36 ± 7	34 ± 6	0.1517	0.341	0.214	0.198
Gender (Male/Female)	71 (59.16%)/49 (40.83%)	64 (53.33%)/56 (46.66%)	41 (51.25%)/39 (48.75%)	0.4890	0.385	0.298	0.450
CD4 Count (cells/μL)	753 ± 101	458 ± 113	399 ± 243	<0.0001	<0.001	<0.001	0.020
Viral Load (copies/mL)	Undetectable	10,537 ± 5056	12,345 ± 8453	<0.0001	<0.001	<0.001	0.015
BMI	26.3 ± 8.1	25.1 ± 4.7	25.6 ± 7.8	0.4057	0.287	0.329	0.414
ART Regimens							
-INSTIs							
Dolutegravir (50 mg/day)	n/a	included	included	n/a	n/a	n/a	n/a
-Bictegravir (in Biktarvy)	n/a	included	included	n/a	n/a	n/a	n/a
-NRTIs							
Tenofovir alafenamide (25 mg/day)	n/a	included	included	n/a	n/a	n/a	n/a
Emtricitabine (200 mg/day)	n/a	included	included	n/a	n/a	n/a	n/a
-Fixed-Dose Combinations							
Biktarvy	n/a	used	used	n/a	n/a	n/a	n/a
TAF/Emtricitabine/Dolutegravir	n/a	used	used	n/a	n/a	n/a	n/a

**Table 2:** Biomarker levels before and 6 months after ART initiation.

Biomarker	Control Group (n = 120)	ART Group (n = 120)	ART + LVDD Group (n = 80)	Global *p*-Value (ANOVA)	Control vs. ART (*p*)	Control vs. ART + LVDD (*p*)	ART vs. ART + LVDD (*p*)
Before ART Initiation							
CRP (mg/L)	1.9 ± 0.8	2.7 ± 1.1	4.5 ± 1.3	<0.001	0.005	<0.001	0.010
IL-6 (pg/mL)	4.2 ± 1.1	5.9 ± 1.8	8.1 ± 2.0	<0.001	0.003	<0.001	0.015
TNF-α (pg/mL)	3.6 ± 1.3	8.2 ± 2.8	13.4 ± 3.1	<0.001	<0.001	<0.001	<0.001
Fibrinogen (mg/dL)	285 ± 48	460 ± 65	485 ± 60	<0.001	<0.001	<0.001	0.020
IL-1β (pg/mL)	3.0 ± 1.2	4.5 ± 1.4	6.2 ± 1.7	<0.001	0.010	<0.001	0.030
IFN-γ (pg/mL)	8.6 ± 2.5	11.2 ± 3.1	14.5 ± 3.3	<0.001	0.002	<0.001	0.012
D-dimer (mg/L)	0.5 ± 0.3	0.8 ± 0.4	1.2 ± 0.5	<0.001	0.005	<0.001	0.025
After 6 Months of ART Initiation							
CRP (mg/L)	1.7 ± 0.7	2.1 ± 0.9	3.8 ± 1.1	<0.001	0.020	<0.001	0.015
IL-6 (pg/mL)	3.9 ± 1.0	4.8 ± 1.7	6.9 ± 1.9	<0.001	0.012	<0.001	0.020
TNF-α (pg/mL)	3.2 ± 1.1	6.5 ± 2.3	11.1 ± 2.8	<0.001	<0.001	<0.001	<0.001
Fibrinogen (mg/dL)	280 ± 45	430 ± 58	460 ± 55	<0.001	<0.001	<0.001	0.035
IL-1β (pg/mL)	2.8 ± 1.0	4.0 ± 1.3	5.6 ± 1.5	<0.001	0.015	<0.001	0.040
IFN-γ (pg/mL)	8.1 ± 2.2	9.6 ± 2.8	12.7 ± 3.0	<0.001	0.005	<0.001	0.018
D-dimer (mg/L)	0.4 ± 0.2	0.7 ± 0.3	1.0 ± 0.4	<0.001	0.008	<0.001	0.025

**Table 3:** Prevalence of gastrointestinal side effects across study groups.

Side Effect	Control Group (n = 120)	ART Group (n = 120)	ART + LVDD Group (n = 80)	Global *p*-Value (ANOVA)	Control vs. ART (*p*)	Control vs. ART + LVDD (*p*)	ART vs. ART + LVDD (*p*)
Nausea	10 (8.33%)	50 (41.66%)	45 (56.25%)	<0.0001	<0.001	<0.001	0.025
Diarrhea	5 (4.16%)	45 (37.5%)	44 (55%)	<0.0001	0.001	0.001	0.010
Abdominal pain	8 (6.66%)	0 (33.33%)	8 (47.5%)	0.0001	0.001	0.001	0.015
Bloating	7 (5.83%)	0 (25%)	8 (35%)	<0.0001	0.001	0.001	0.045

**Table 4:** Cardiac function parameters in HIV-positive patients on ART with Grade 1 and Grade 2 left ventricular diastolic dysfunction (LVDD).

Parameter	Group 3 (HIV + ART + LVDD Grade 1)	Group 3 (HIV + ART + LVDD Grade 2)	*p*-Value
Number of Patients	45	35	-
Mean Age (years)	52 ± 8	54 ± 7	0.31
E/A Ratio	0.8 ± 0.2	1.5 ± 0.3	<0.01
E/e’ Ratio	8.5 ± 1.2	12.3 ± 1.5	<0.001
Left Atrial Volume Index (mL/m²)	29 ± 4	35 ± 6	<0.01
Ejection Fraction (%)	62 ± 5	58 ± 6	0.02
Ventricular Filling Pressure	Normal	Moderately elevated	-
Clinical Symptoms	25%	57%	<0.01
CRP (mg/L)	3.2 ± 1.1	3.8 ± 1.1	0.02
IL-6 (pg/mL)	4.8 ± 1.7	6.9 ±1.9	0.01
TNF-α (pg/mL)	6.5 ± 2.3	11.1 ± 2.8	<0.001
Fibrinogen (mg/dL)	430 ± 58	460 ± 55	0.035
Nausea (%)	12 (26.66%)	20 (57.14%)	0.011
Diarrhea (%)	10 (22.22%)	16 (45.71%)	0.032
Abdominal Pain (%)	7 (15.55%)	12 (34.28%)	0.066

**Table 5:** Correlation between inflammatory biomarkers and gastrointestinal side effects.

Biomarker	Nausea	Diarrhea	Abdominal Pain	Bloating
C-reactive protein (CRP)	0.65	0.60	0.55	0.50
Interleukin-6 (IL-6)	0.62	0.58	0.54	0.48
Tumor necrosis factor-alpha (TNF-α)	0.68	0.63	0.57	0.52
Fibrinogen	0.60	0.55	0.53	0.45

**Table 6:** Multivariate logistic regression analysis of predictors for gastrointestinal side effects.

Variable	Odds Ratio (OR)	95% Confidence Interval (CI)	*p*-Value
CRP	1.75	1.40–2.20	<0.0001
IL-6	1.68	1.37–2.10	<0.0001
TNF-α	1.82	1.45–2.31	<0.0001
Fibrinogen	1.60	1.30–2.00	0.003 *
IL-1β (pg/mL)	1.25	1.06–1.51	0.007 *
IFN-γ (pg/mL)	1.61	1.29–1.84	<0.001 *
D-dimer (mg/L)	1.34	1.14–1.64	<0.001 *
Age	1.05	1.02–1.08	0.010 *
BMI	1.10	1.05–1.20	0.008 *
Gender (female)	1.12	0.95–1.33	0.181

**Table 7:** Comparison of logistic regression analysis results for predictors of gastrointestinal symptoms.

Predictor	Odds Ratio (HIV+)	95% CI (HIV+)	*p*-Value (HIV+)	Odds Ratio (HIV + LVDD)	95% CI (HIV + LVDD)	*p*-Value (HIV + LVDD)
CRP	1.75	1.40–2.20	<0.0001	2.10	1.75–2.60	<0.0001
IL-6	1.68	1.37–2.10	<0.0001	1.90	1.55–2.40	<0.0001
TNF-α	1.82	1.45–2.31	<0.0001	2.25	1.85–2.70	<0.0001
Fibrinogen	1.60	1.30–2.00	0.003	1.80	1.45–2.20	0.002
IL-1β	1.25	1.06–1.51	0.007	1.30	1.10–1.60	0.006
IFN-γ	1.61	1.29–1.84	<0.001	1.75	1.40–2.10	<0.001
D-dimer	1.34	1.14–1.64	<0.001	1.50	1.20–1.80	<0.001
Age	1.05	1.02–1.08	0.010	1.08	1.03–1.15	0.008
BMI	1.10	1.05–1.20	0.008	1.15	1.07–1.25	0.006
Gender (female)	1.12	0.95–1.33	0.180	1.20	1.00–1.45	0.078

## Data Availability

The datasets used and/or analyzed during the current study are available from the first author.
